# Sedimentary and diagenetic characteristics of the Z21 field in the Huizhou depression, Pearl River Mouth basin, South China Sea

**DOI:** 10.1038/s41598-022-09532-y

**Published:** 2022-03-31

**Authors:** Longlong Liu, Hongzhi Dong, Yinjiao Wu, Ronghua Fan, Zhongqiang Sun, Rihui Huang, Jinliang Zhang

**Affiliations:** 1 School of Geographical Sciences, Lingnan Normal University, Zhanjiang, 524048 China; 2 College of Earth Science and Engineering, Shandong University of Science and Technology, Qingdao, 266510 China; 3 No.2 Oil Production Plant, PetroChina Qinghai Oilfield Company, Dunhuang, 736202 China; 4 College of Resources Science and Technology, Beijing Normal University, Beijing, 100875 China

**Keywords:** Geology, Stratigraphy

## Abstract

The Huizhou Depression in the Pearl River Mouth basin has prospective hydrocarbon potential, with Miocene sandstones as its main oil and gas-bearing reservoir. The sandstones in Miocene formation of the Z21 offshore oil–gas field composed of medium-grained, moderately sorted subarkose and lithic arkose. In this study, a total of six depositional lithofacies, namely Massive fine- to medium-grained sandstone (Sm), ripple cross-laminated fine-grained sandstone (Sr), parallel-laminated siltstone and claystone (Fl), lenticular siltstone (Sl), parallel-bedded fine-grained sandstone (Sp), wavy laminated siltstone (Sw), and two depositional systems, namely nearshore sand bar (SB) and sand sheet (SS) were identified based on core observations and seismic study. Distributions of the porosity (13.9%) and permeability (35.8 mD) reveal that the Miocene sandstones have characteristics of low porosity and low permeability, with high heterogeneity. The sedimentary system, primary texture and diagenesis jointly control the reservoir quality. Sandstones with sand bars as well coarse-grained tend to exhibit a higher quality. Mechanical compaction and calcite (average 6.81%) cementation are the major determinants to reductions in porosity and permeability. The total clay minerals (average 5.27%) generally lead to reduction of porosity, whereas chlorite coatings and illite within a certain content range may enhance the preservation of porosity in eodiagenesis. Dissolution of feldspar and debris contribute significantly to improving the reservoir quality.

## Introduction

**Figure 1:**
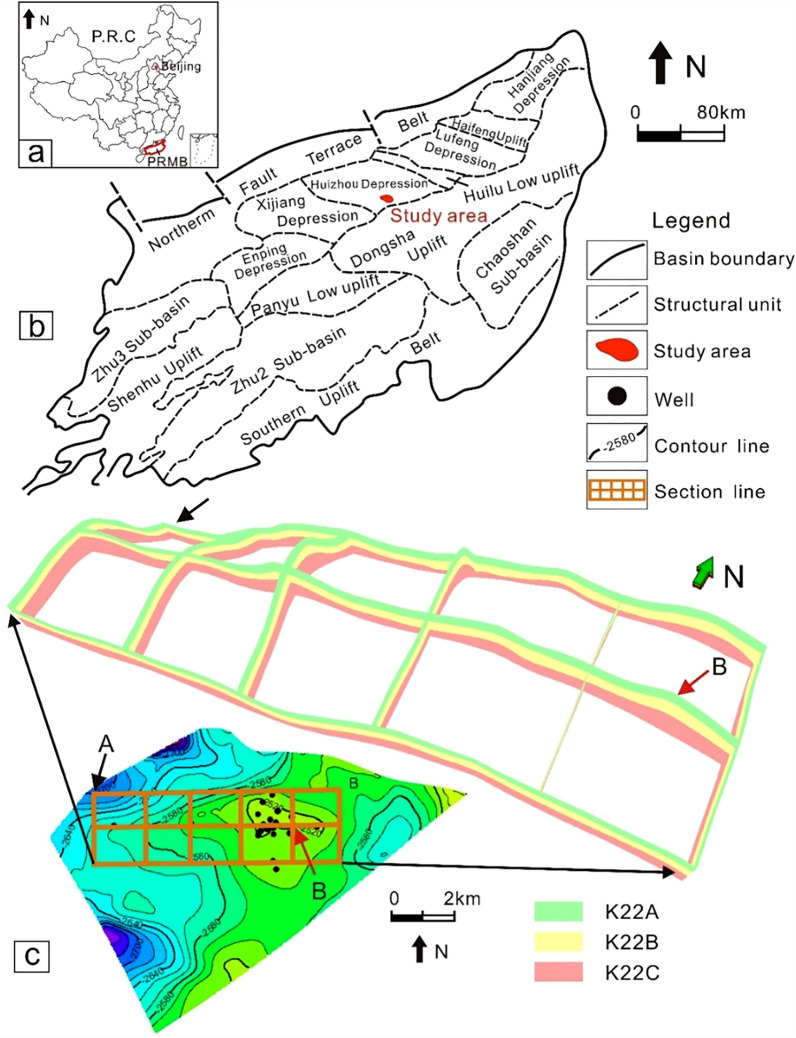
(**a**) Map of the People’ Republic of China and location of the PRMB^14^. (**b**) Structural map of the PRMB, showing different sub-structural units in the basin^14^. As shown in the figure, the PRMB is a complicated structural unit including multiple depressions and sub-basins as prospective accumulative space for hydrocarbon. (**c**) Three-dimensional fence diagram of the studied strata. “A” refers to a structural low, “B” refers to a structural high. K22A, K22B and K22C are three subdivided layers from K22 layer. The red arrows refer to the structural high, the black arrow refers to the structural low. Figure (**a,b)** are modified from Liu et al.^14^.

Ocean energy resources play a significant role in national interests and people’s livelihoods. The development of ocean energy resources not only guarantees China’s energy security but also reflects the national sustainable capacity^1^. Hydrocarbon resources, as essential types of ocean energy resource, have drawn increasing attention with respect to prospectivity, exploration, exploitation and other aspects. According to the results of the national oil and gas resource evaluation, the geological reserves of offshore oil and gas in China are 1.074 × 10^10^ t and 8.1 × 10^12^ m^3^, respectively^2^. As with the Bohai Bay Basin^3^ and the East China Sea Shelf Basin^4^, the Pearl River Mouth Basin (PRMB) in the South China Sea (Fig. 1a) contains agates and is characterized by abundant petroleum resources and other natural resources^5,6^. The Huizhou depression, one of the many potential depressions in the PRMB (Fig. 1b), has been studied by many geologists in terms of the source rock^7^, stratigraphic sequence^8^, depositional system^9–11^ and other aspects. The Huizhou depression is an important area that contains abundant oil and gas accumulation, with a total gas reserve of 20.87 × 10^8^ m^3^ and a proven reserve of 6.03 × 10^8^ m^3^ ^12,13^. The offshore Z21 gas field (Fig. 1b,c), was discovered in 1990 and is the only gas field in the Huizhou depression. To date, there are a total of 17 development wells and 3 exploratory wells in this field.

Reservoir connectivity and reservoir quality are two issues of concern for the gas field. The reservoir connectivity of the Z21 field was discussed via a qualitative sequence-related method by Ding et al.^10^ and via a quantitative method by Liu et al.^14^. For the sake of future development and production in the field, it is critical to study the factors controlling reservoir quality. This paper aims to study the sedimentary features and diagenetic processes of the Miocene Zhujian Formation in the Z21 field and to quantitatively analyse the factors controlling reservoir quality for the sake of a comprehensive understanding of reservoir characteristics. Moreover, petroleum accumulation characteristics are qualitatively analysed based on a reservoir quality study.

**Figure 2:**
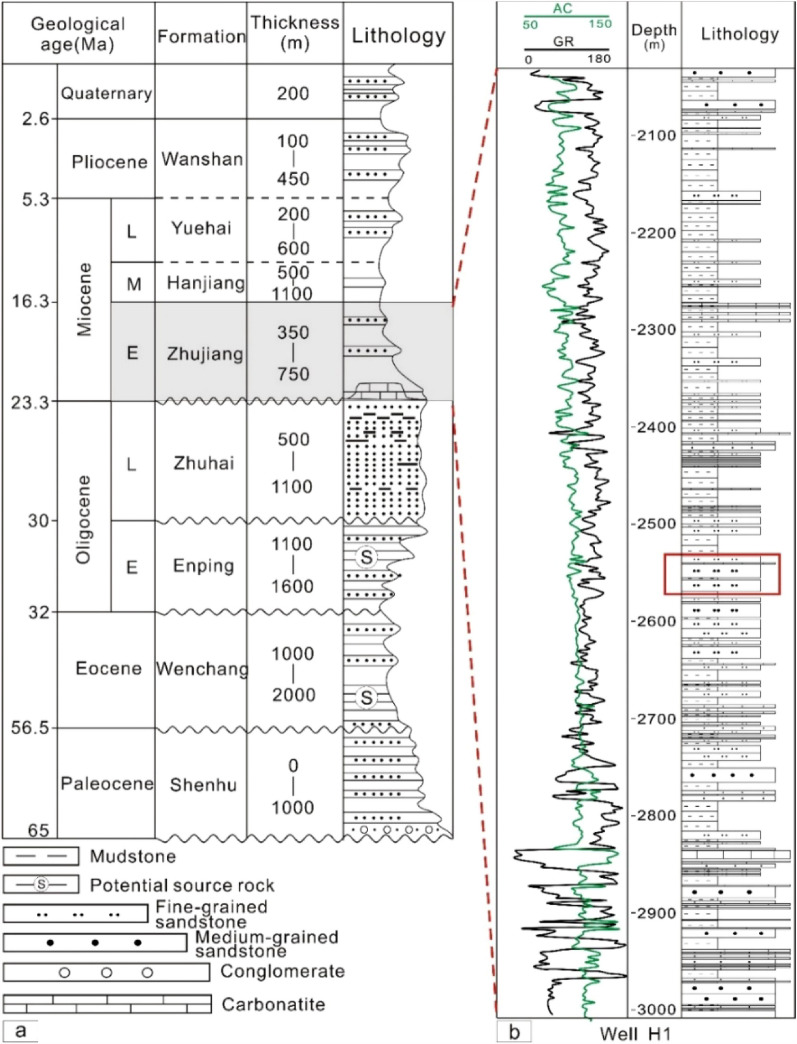
(**a**) Stratigraphic units of the PRMB (Modified after Chen and Pei^17^and Robison et al.^18^). Notice two sets of potential source rocks in Enping and Wenchang Formations in terms of thickness and continuity. (**b**) Detailed stratigraphic column of well H1. Varying vertical lithofacies imply strong depositional heterogeneity for the reservoirs. The K22 layer is shown in the red box^14^.

The Pearl River Mouth Basin is an offshore extension of the South China continent (Fig. 1a) with a length of approximately 800 km, widths of 100–300 km and an area of 27 km^2^. Its depth varies from dozens of metres to more than 3000 m^15,16^. The Huizhou depression lies in the mid-eastern region of the PRMB and is limited by the North Fault Terrace in the north and the Dongsha uplift in the south. Its eastern and western areas are the Huilu Low uplift and Xi-jiang depression, respectively (Fig. 1b). The study area, the Z21 oil–gas field, lies in the southern Huizhou depression adjacent to the Dongsha uplift (Fig. 1b). In a structural sense, structural highs are developed in the east, and structural lows are in the northwest, southwest and northeast (Fig. 1c). The fence diagram from the three-dimensional structural model provides a visual perspective to understand the structural features (Fig. 1c). The target reservoir, the K22 layer, which lies in the middle of the Zhujiang Formation (Fig. 2), is the main production interval of the Z21 oil–gas field.

From Quaternary to Paleocene strata, the basin contains eight formations from top to bottom: Wanshan (WS), Yuehai (YH), Hanjiang (HJ), Zhujiang (ZJ), Zhuhai (ZH), Enping (EP), Wenchang (WC) and Shenhu (SH)^14,17,18^ (Fig. 2a). In the Huizhou depression, the main sandstone reservoirs are the ZH and ZJ Formations. In the lower ZJ Formation, a set of carbonate platform facies are widely developed, whereas in the middle and upper parts of the ZJ Formation, two sets of fine- to medium-grained sandstones are widely deposited, forming the principal reservoir of the Huizhou depression (Fig. 2b).

The deposits of the ZH Formation, characterized by a thickness range of 500–1100 m, vary from continental delta to marine deltaic facies^8,19^ and also consist of sediments dominated by tides^20^. In terms of the stratigraphic sequence, the short-term, middle-term and long-term cycles were studied by Cheng et al.^21^ and Wei et al.^8^. Based on seismic technology, such as root mean square amplitudes, with the assistance of sedimentology and sequence stratigraphy, favourable areas for prospecting reserves in the Huizhou depression were predicted^16,21,22^.

It is common knowledge that the WC Formation and EP Formation played an essential role in the hydrocarbon accumulation in the Huizhou depression^7,23^. The EP Formation is characterized by two thin sets of sandstones in the lower section and a thick set of dark-coloured mudstones in the upper section (Fig. 2a), which are viewed as potential source rocks^14,24^. The underlying WC Formation unconformably contacts the EP Formation, and its lower strata are dominated by a thick set of semideep to deep lacustrine dark grey mudstones (Fig. 2a), which are considered the essential source rocks in the Huizhou depression^19,22^. In short, the dark-coloured mudstones of the WC and EP Formations are important source rocks for hydrocarbon accumulations in the Huizhou depression^14^.

## Materials and methods

Depositional systems, including depositional lithofacies and systems, were analysed based on sedimentary structure, texture, grain size, rock colour, seismic data, stratigraphic characteristics and other preexisting geologic knowledge. The grain size (AD) is calculated by the formula ϕ =  − log_2_ D, (D: particle diameter)^25^. The sorting (S_o_) is the sediment sorting degree parameter, which is calculated by the following formula: S_0_ = P_25_/P_75_ (P_25_: particle diameter of the cumulative particle content at 25%; P_75_: particle diameter of the cumulative particle content at 75%; good: 1–2.5; moderate: 2.5–4; poor: > 4).

A total of 40 representative core samples in the Miocene reservoirs were collected from 5 cored wells in the Huizhou depression. To determine the pore types and diagenetic minerals of the Zhujiang Formation, 20 thin sections were made after each core sample was vacuum impregnated with blue epoxy resin. Twenty core samples were used for scanning electron microscopy (SEM) to identify diagenetic minerals and pore types using HITACHI H600 equipment equipped with a LinKQX-200 energy dispersive spectrometer operating at an accelerating voltage of 20 kV and an emission current of 9000 or 9400 nA. The point counting method (200–300 points per thin section) was applied to analyse rock constituents, such as quartz, feldspar, rock fragments and cements. XRD (X-ray diffraction) was conducted using a Rigaku D/MAX-2400 X-ray diffractometer at the Rock-Mineral Preparation and Analysis Lab of the Institute of Geology and Geophysics, Chinese Academy of Sciences, Beijing, China. The porosity and permeability were collected from the Shenzhen Branch of the CNOOC (China National Offshore Oil Corporation).

## Results

### Mineral composition and texture

**Figure 3:**
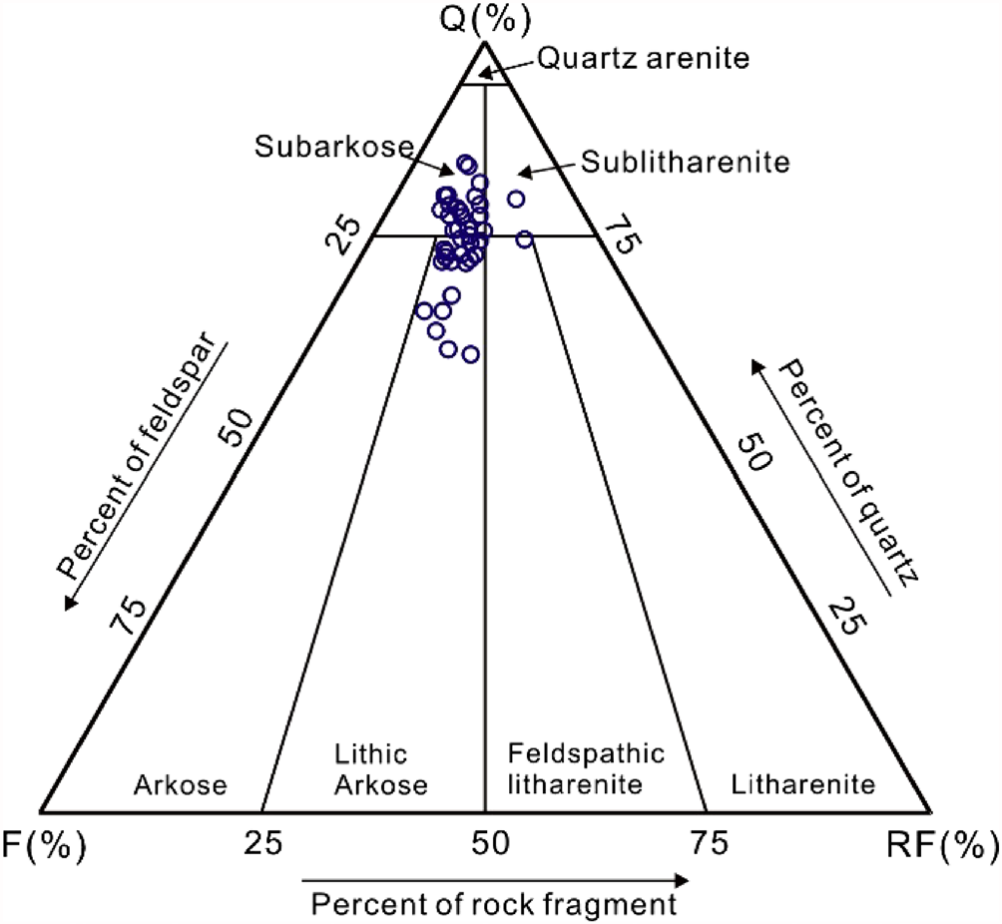
Ternary diagram exhibiting the framework grain composition of the Miocene Zhujiang Formation in Z21 oil–gas field in Huizhou depression. Q = quartz, F = feldspar, RF = rock fragments. This figure shows that the studied interval is dominated by subarkose and lithic arkose.

The detrital mineralogy of the Miocene Zhujiang sandstones is dominated by quartz (range of 60.5–85.9 vol%, average of 75.7 vol%), followed by feldspar (range of 4.7–24.1 vol%, average of 14.5 vol%), and rock fragments (range of 4.6–18.6 vol%, average of 9.9 vol%) (Table 1). Therefore, the reservoir sandstones of the Z21 oil–gas field are mainly classified as subarkose and lithic arkose in the average framework composition of Q_76_F_14_R_10_ based on the classification by Folk^25^ (Fig. 3).

**Table 1:** Summary point-counting data exhibiting the detrital composition of the Miocene Zhujiang Formation in Z21 oil–gas field.

Well	Depth (m)	AD (mm)	Sorting (S_o_)	Framework composition (%)				Diagenetic minerals				Clay (%)	Ф (%)	K (mD)	Pt (%)
				Q	F	R	Mi	Qc	Cal	Daw	Py				
H3	2702.8	0.4	Poorly	58.0	14.0	7.0	/	√	6.0	/	/	9.0	19.5	43.0	6.0
H3	2582.5	0.2	Well	59.0	6.0	4.0	/	/	18.0	√	1.0	9.0	4.0	46.0	3.0
H3	2582.3	0.3	Moderately	62.0	10.0	10.0	√	0.1	4.8	√	√	5.0	19.6	16.0	8.0
H3	2582.0	0.1	Well	59.0	14.0	8.0	0.5	0.1	10.4	√	/	6.0	5.4	0.4	2.0
H3	2581.5	1.0	Poorly	62.0	10.0	6.0	0.1	/	4.6	/	/	8.0	20.1	7.3	9.0
H3	2581.0	0.4	Moderately	63.0	12.0	4.0	√	/	5.1	/	√	6.0	14.3	6.2	10.0
H3	2579.9	0.5	Poorly	60.0	11.0	5.0	1.0	0.5	8.5	2.0	/	5.0	19.0	14.0	7.0
H1	2567.5	0.4	Well	54.0	10.0	6.0	√	√	25.0	/	1.0	/	/	/	4.0
H1	2554.7	0.2	Well	41.0	7.0	3.0	√	/	45.0	/	1.0	/	/	/	3.0
H1	2553.3	0.1	Well	70.0	7.0	7.0	2.0	√	/	/	/	13.0	/	/	2.0
H1	2551.8	0.2	Moderately	66.0	8.0	7.0	2.0	/	1.0	/	1.0	13.0	/	/	2.0
H1	2525.6	0.3	Well	52.0	18.0	16.0	3.0	0.1	/	/		6.0	/	/	5.0
H1	2524.1	0.3	Well	74.0	9.0	9.0	3.0	0.5	/	/	0.5	4.0	/	/	1.0
H1	2523.5	1.0	Well	69.0	4.0	12.0	1.0	/	/	/	/	4.0	/	/	10.0
H1	2521.5	0.3	Moderately	67.0	7.0	4.0	3.0	√	/	/	/	4.0	/	/	1.0
H1	2520.8	0.5	Moderately	52.0	20.0	13.0	0.1	1.0	1.0	/	1.0	6.0	/	/	6.0
H1	2520.2	0.4	Well	56.0	21.0	11.0	√	√	0.5	0.5	2.0	2.0	/	/	7.0
H1	2519.9	0.2	Poorly	75.0	10.0	10.0	√	/	1.0	√	1.0	2.0	/	/	1.0
H1	2519.8	0.3	Well	72.0	6.0	17.0	1.0	/	/	/	/	2.0	/	/	2.0
H1	2519.7	0.3	Well	64.0	15.0	7.0	0.3	√	√	0.5	√	6.0	/	/	7.0
H1	2518.8	1.0	Poorly	71.0	12.0	4.0	0.1	/	2.0	1.0	√	2.0	/	/	8.0
H1	2518.5	0.5	Well	67.0	11.0	7.0	1.0	√	/	/	/	3.0	/	/	11.0
H1	2518.2	0.5	Moderately	61.0	13.0	10.0	2.0	/	1.0	√	1.0	3.0	/	/	9.0
H1	2517.3	0.5	Moderately	55.0	18.0	10.0	2.0	√	/	/	√	5.0	/	/	10.0
H1	2517.1	0.2	Well	60.0	14.0	7.0	3.0	/	2.0	√	/	9.0	/	/	5.0
H1	2516.8	0.2	Well	55.0	20.0	8.0	2.0	√	1.0	/	1.0	8.0	/	/	5.0
H1	2516.4	1.0	Moderately	70.0	10.0	11.0	1.0	0.5	/	0.5	/	1.0	/	/	6.0
H1	2515.8	0.5	Poorly	67.0	13.0	7.0	1.0	/	1.0	√	1.0	1.0	/	/	9.0
H1	2515.6	0.4	Well	68.0	11.0	6.0	1.0	√	1.0	/	/	2.0	/	/	11.0
H1	2515.1	0.3	Moderately	63.0	12.0	8.0	1.0	/	1.0	/	1.0	3.0	/	/	11.0
H1	2515.0	0.6	Well	66.0	11.0	8.5	1.0	/	2.0	/	0.5	3.0	/	/	8.0
H1	2519.6	0.5	Poorly	56.0	16.0	10.0	3.0	/	1.0	1.0	/	4.0	/	/	9.0
H1	2519.3	0.4	Poorly	59.0	15.0	7.0	2.0	/	2.0	/	1.0	4.0	/	/	10.0
H2	2436.6	0.6	Moderately	61.0	11.0	9.0	/	0.0	1.8	0.5	1.0	3.0	20.8	210.2	13.0
H2	2433.8	0.3	Well	49.0	8.0	3.0	√	0.5	25.4	1.0	√	12.0	3.4	0.0	1.0
H2	2431.7	0.5	Moderately	58.0	10.0	8.0	0.5	/	12.5	/	1.0	10.0	10.8	0.2	6.0
H2	2429.7	0.1	Well	59.0	11.0	10.0	/	/	12.0	/	/	6.0	7.6	1.4	2.0
H6-1	2039.9	0.1	Well	60.0	12.0	10.0	0.5	√	0.5	2.0	1.0	4.0	18.0	186.0	10.0
H6-1	2036.5	0.4	Poorly	62.0	13.0	9.0	1.5	/	0.5	/	/	2.0	13.4	230.0	12.0

*AD* average diameter of grains, *Q* quartz, *F* feldspar, *R* rock fragments, *Mi* Mica, *Qc* silica cementation, *Cal* calcite, *Daw* dawsonite, *Py* pyrite, *Ф* measured porosity, *K* measured permeability, *Pt* thin section porosity, /: without; √: trace.

The Miocene sandstones are composed of grains ranging from 0.1 to 1 mm in diameter, with an average of 0.39 mm. The sandstones feature fine- (accounting for 28.6%) to coarse-grained (accounting for 21.4%) sandstones and are dominated by medium-grained sandstones (accounting for 50%). The percentages of poorly sorted, moderately sorted and well sorted sandstones are 23.1%, 28.2% and 48.7%, respectively (Table 1).

### Depositional environment

#### Lithofacies

**Figure 4:**
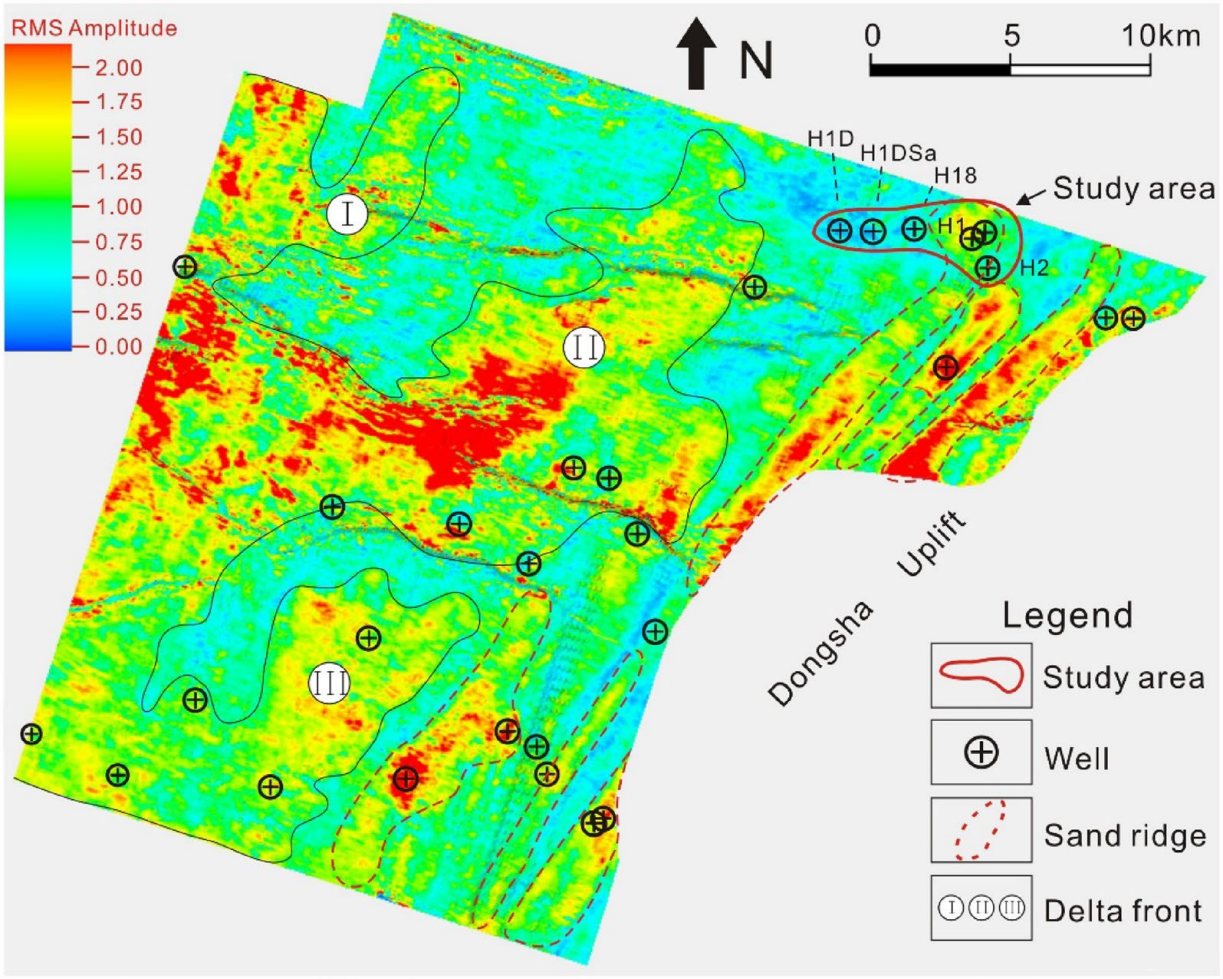
Seismic distribution model sandstone and mudstone of K22 layer, obtained from geostatistical inversion (After CNOOC)^14^. This inversion profile is based on high-resolution seismic data and logging data. The stochastic obvious distinctions presented by different values indicate clustering of sandstone (high values) and mudstone (low values).

**Figure 5:**
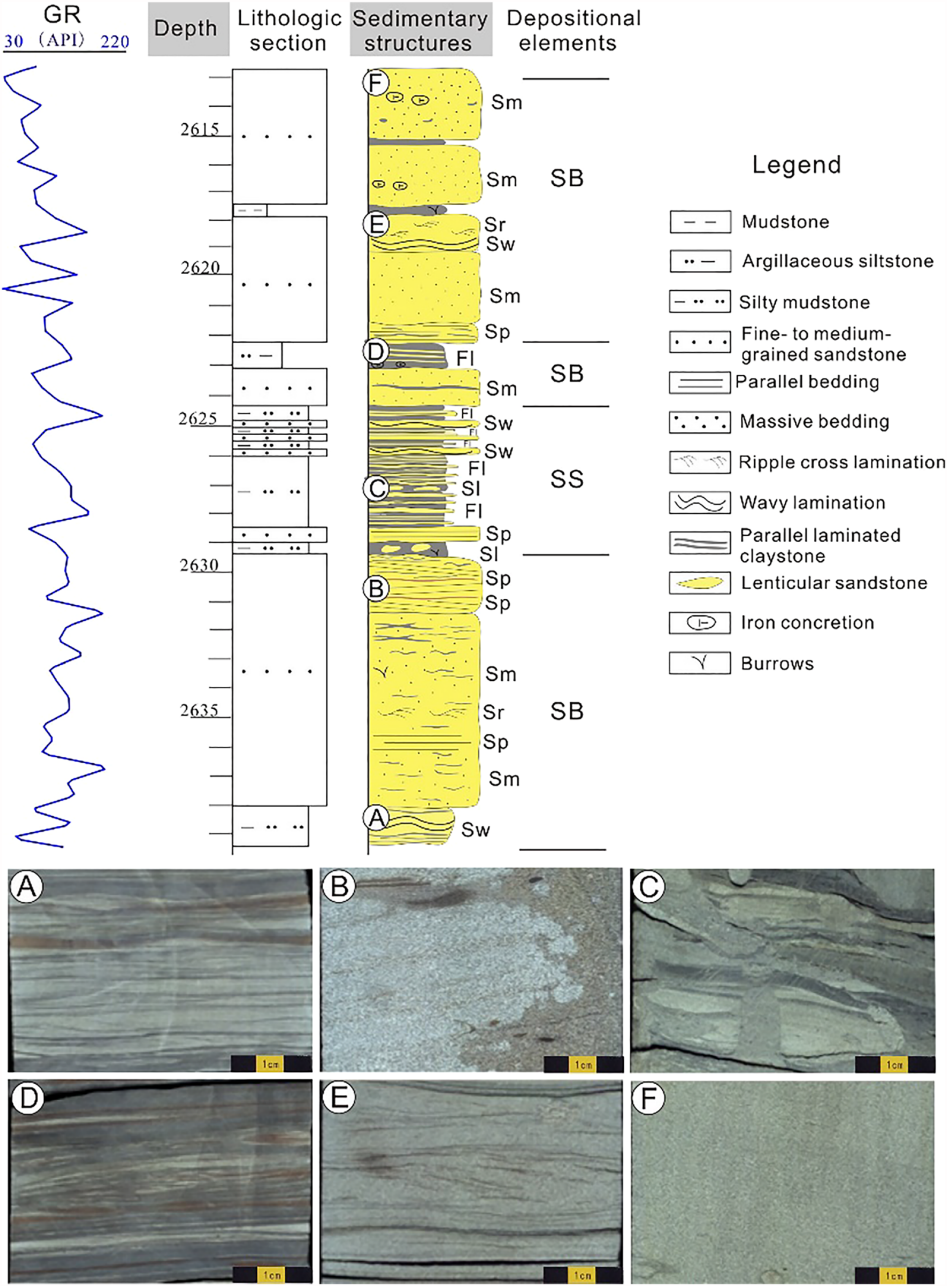
Single well section description of well H2. Various sedimentary structures indicate complicated hydrodynamic environment. Sm, SP, Sr, Sl, Sw and Fl represent 6 different lithofacies. SB and SS represent 2 different depositional systems. See detailed information of these acronyms in the text.

Deposits in the Z21 oil-gas field were considered sand ridges located far from the delta front, dominated by wave and shore currents (Fig. 4), which is consistent with previous studies^11,14^. A total of 6 depositional lithofacies were identified (Fig. 5), namely, massive fine- to medium-grained sandstone (Sm, Fig. 5A), ripple cross-laminated fine-grained sandstone (Sr, Fig. 5B), parallel-laminated siltstone and claystone (Fl, Fig. 5C), lenticular siltstone (Sl, Fig. 5D), parallel-bedded fine-grained sandstone (Sp, Fig. 5E), and wavy laminated siltstone (Sw, Fig. 5F). Brief descriptions of these six lithofacies are given as follows.

1. Massive fine- to medium-grained sandstone (Sm). This facies is generally fine- to medium-grained, 1 to 3 m thick, and light grey in colour (Fig. 5A). This facies is tight, well-sorted, siliceous-cemented, slightly calcareous and occasionally embedded by vertical burrows or tiny muddy strips (Fig. 5A). It is interpreted as deposits that rapidly formed during the establishment of offshore sand bars with current and wave energies^26^.
2. Ripple cross-laminated fine-grained sandstone (Sr). This facies is usually found within a short interval (less than 0.5 m). Obvious soft sediment deformation structures are occasionally observed. Isolated ferric concretions are occasionally observed within this facies (Fig. 5B). In places, small-scale burrows are developed. This lithofacies is interpreted as the migration of ripples in fluctuating currents, indicating a lower energy regime^27,28^.
3. Parallel-laminated siltstone and claystone (Fl). This facies consists of laminated siltstone or “colourful” claystone units interbedded with millimetre-thick siltstone laminations (Fig. 5C). Both the upper and lower contacts with facies Sm, Sw or Sl are sharp (Fig. 5). Common structures include vertical and lateral bioturbations, calcareous concretions and iron nodules. The fresh surfaces of the interlaminated siltstone are off-white in most instances, whereas they are light red or brown on the weathered surfaces. This lithofacies is interpreted as deposits formed in a laminar plug flow^29^.
4. Lenticular siltstone (Sl). This lithofacies makes up a large proportion of the cores from the K22 reservoir set. Figure 5D shows the typical lenticular siltstone facies in cores from well H2. Depositional structures within this facies are complex”, including wavy bedding, flaser bedding, small-scale ripple bedding and lenticular bedding (Fig. 5D). The shapes of the individual lenticular siltstone bodies are quite different, and the insides of the lenticular bodies are commonly sparsely filled with tiny muddy ripple laminations (Fig. 5D). Decimetre-thick wavy strips that are brown or light brown in colour are commonly interstratified within this lithofacies. Lenticular siltstone is generally related to the transformation of delta front bedforms, which benefit from continuous or intermittent waves and coastal currents^29,30^.
5. Parallel-bedded fine-grained sandstone (Sp). Most of this facies is approximately horizontally bedded, with interbedded thin muddy laminations with random unequal separation distances (Fig. 5E). This facies is several centimetres to 1.5 m thick and light grey or silver grey in colour. In many instances, clear parting lineations are observed in cores, and muddy laminations expand because of moisture absorption. In some places, yellowish brown laminations rich in ferric ions are developed within this facies. This lithofacies is interpreted to represent high sedimentation rates during the gap between the rise and fall of the waves^11,30^.
6. Wavy laminated siltstone (Sw). This lithofacies accounts for a small proportion of all the lithofacies. It is characterized by a thin thickness (less than 0.2 m) and poor continuity due to interruptions of other lithofacies (e.g., Sr or Sp). Isolated calcareous concretions or 0.01 m or thinner laminations that are rich in iron material are occasionally developed (Fig. 5F). In general, both the crests and troughs of the waves are relatively gentle (Fig. 5F). This lithofacies is interpreted as a waning current deposit in a tidal setting^29^.

#### Depositional system

Two primary depositional systems characterize the K22 interval: nearshore sand bar (SB) and sand sheet (SS). The identification of these two systems is mainly based on lithofacies, sedimentary structures, and sequences. The description and interpretation of the two systems are briefly discussed below.

1. Depositional system SB**.** As shown in Fig. 4, SB varies from 1.5 to 5 m in thickness and has multiple facies associations. The sandstone:mudstone ratio is high, and the continuity of SB is generally interrupted by thin units of facies Fl or Sl. Both the upper and lower contacts with facies Fl and Sl are gradual and/or sharp. In many places, especially within the K22A layer, the massive sandstone of SB is well developed (Fig. 5). It is common that isolated iron concretions that are elliptical, spherical, bamboo-shaped or finger-shaped, are developed within this system. In places, severe muddy rip-up clasts are dispersed within the silty facies, Sm. Bioturbation is occasionally found within facies Sw, Sl or Fl in this succession.

SB represents the deposits of the delta front bedform, which was deposited by waves and currents^11^. In this paper, recognition of SB in the plane with the assistance of seismic data (Fig. 4) is in accordance with previous works^8,10,31^. As shown in Fig. 3, the west and southwest areas of the study area, which are marked by three zones, namely, I, II and III, have been widely acknowledged as delta front depositional systems, whereas the main zone of the Z21 field is involved in the sand ridges, which are distributed far from the delta lobe^10,32^. As the delta front advances towards the sea basin, the preexisting coarse-grained deposits are continuously transported away from the prodelta zone by the force of waves and then deposited. If the sediment supply is sufficient, the redeposited sand is transported again for a certain distance until the wave force vanishes^14^. The accumulation of the reworked deposits forms a series of sand ridges that are approximately parallel to the shoreline^14^. The amplitude of SB is high, and the system obviously has an NNE trend (Fig. 4).

2. Depositional systems SS. The SS system is characterized by an individual set of argillaceous siltstones that are less than 1 m thick and are interbedded with mudstone (Fig. 5). Facies Fl and Sl are well developed within this succession with abundant burrows. Iron concretions with different shapes are also occasionally found in facies Fl and Sl. Other sedimentary structures, including small-scale ripple laminates, soft deformation structures and calcareous concretions, are observed in places. In general, SS is dominated by muddy deposits with a high mudstone:sandstone ratio.

SS is interpreted as the result of the transfer and reconstruction of the large delta front bedform by waves and coastal currents^11,33^. According to the amplitude slice of the K22 set, the amplitude of the SS is relatively low, and its geometry is sheet-like (Fig. 4). Within the SS, the flow energy changes frequently, resulting in various sedimentary structures (Fig. 5). According to the seismic inversion, the western reservoir at well H18 could be classified as an SS depositional zone, whereas well H18 is possibly distributed at the margin of the SB or within the SS (Fig. 4).

### Pore classification and physical properties

**Figure 6:**
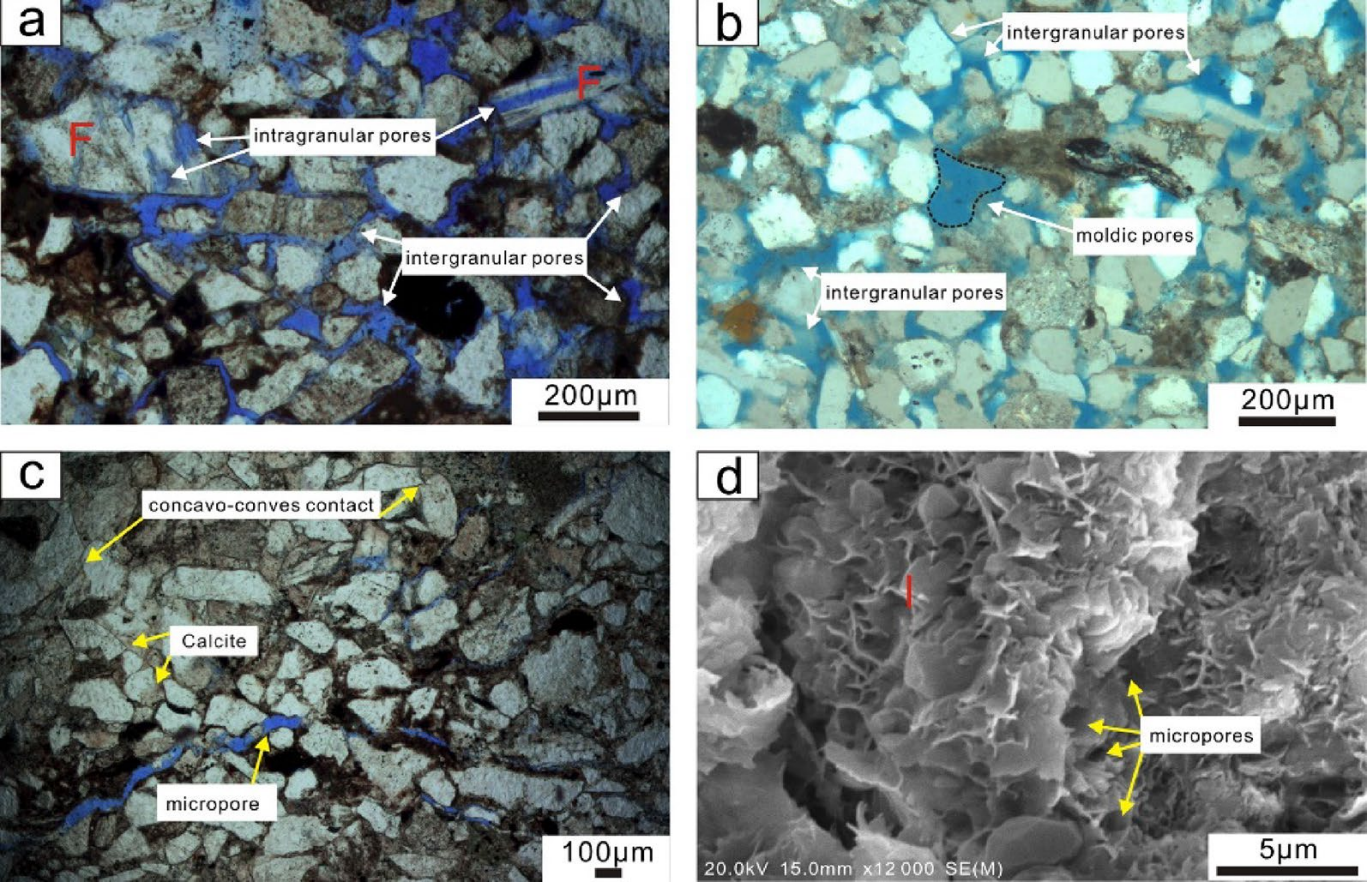
Thin section photomicrographs and SEM images showing details of grain and pore modifications. (**a**) Primary intergranular pores and secondary dissolution intragranular pores resulting from dissolution of feldspars under the microscope, well H2, 2436.6 m, plane-polarized light (PPL); (**b**) Primary intergranular pores and moldic pores resulting from complete dissolution of grains, in well H3, 2702.82 m, PPL. (**c**) Micro-fractures distributed between directionally arranged grains, concavo-conves contact are frequent in this view, well H2, 2433.85 m, PPL; (**d**) Intercrystalline micropores within honeycomb-like illite cements, widths of this types of micropores are usually less than 5 μm, well H4, 2621.5 m, SEM. F = feldspar, I = illite.

According to the classification by Schmidt and Mcdonald^34^, based on the thin section analysis and SEM images, three types of pores, namely, residual primary intergranular pores (Fig. 6a,b), secondary dissolution pores (Fig. 6a,b), and micropores (Fig. 6c,d), are distinguished in the Miocene reservoir sandstones.

The point counting results of the thin sections show residual primary intergranular pores accounting for 82.5% of the total pores, indicating that the pores of the Miocene sandstones in the ZJ Formation are dominated by this pore type. Through observation, the residual primary intergranular pores mainly occur under the condition that there is little to no cement or matrix blocking the space between detrital grains (Fig. 6a,b). The secondary dissolution pores are further classified into intragranular pores (Fig. 6a) and intergranular dissolution pores (Fig. 6b, moldic pores). The former were generally produced by the incomplete dissolution of unstable grains, such as feldspar and debris, whereas the latter were mainly generated from the dissolution of the edges of detrital grains. The moldic pores were the result of the complete dissolution of detrital grains (Fig. 6b). There are two kinds of micropores developed within the ZJ Formation: microfractures (Fig. 6c) and intercrystalline micropores (Fig. 6d). The microfractures are mostly observed within the intervals within directionally arranged grains (Fig. 6c), whereas the intercrystalline micropores are mainly developed within intervals of illitic clays (Fig. 6d).

**Figure 7:**
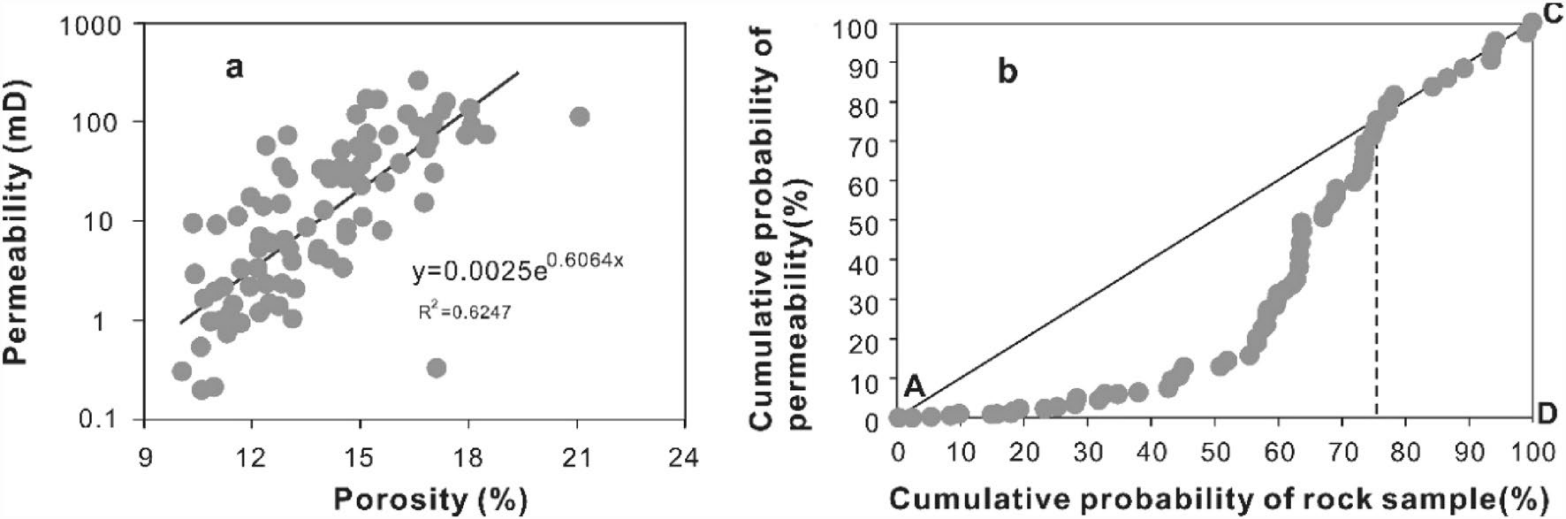
(**a**) Relationship between porosity and permeability. (**b**) Lorenz plot of permeability of K22 set in Z21 oil–gas field. Strong reservoir heterogeneity is indicated by this figure.

The porosity of the target formation varies from 10.1 to 20.1%, with a mean value of 13.9%. The minimum permeability value is 0.2 mD, the maximum value is 230 mD, and the average value is 35.8 mD. The porosity and permeability are characterized by a positive linear relationship (Fig. 7a). The Lorenz plot of permeability in Fig. 7b aims to describe the reservoir heterogeneity. For a homogeneous reservoir, the Lorenz plot is characterized by a straight-line AC, unlike a heterogeneous reservoir^35,36^. The Lorenz plot of permeability for the Z21 oil–gas field is an arc within 0–75.21% of the cumulative probability of the rock sample range, indicating strong reservoir heterogeneity.

### Diagenesis and diagenetic mineralogy

#### Compaction

**Figure 8:**
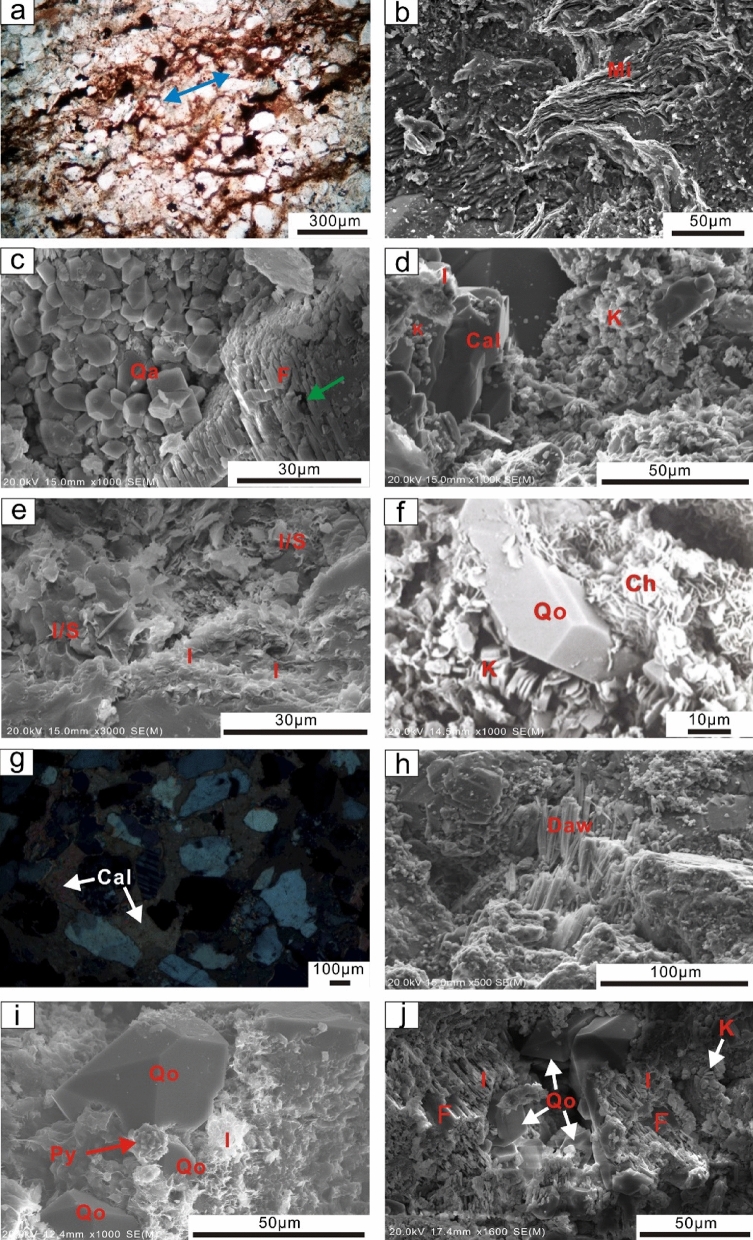
Optical thin sections and SEM images showing (**a**) directional arrangement of framework grains caused by mechanical compaction, approximately in the direction shown by the blue arrow, well H4, 2530.5 m, PPL; (**b**) deformed rock fragment, probably micas, well H3, 2560.8 m, SEM; (**c**) authigenic quartz cements adjacent to dissolved feldspar with obvious intragranular pores (pointed by a green arrow), well H2, 2536.6 m, SEM; (**d**) vermiform kaolinite accompanied by the coprecipitation of calcite in the primary residual intergranular pores, well H3, 2702.8 m, SEM; (**e**) fibrous illite, partially converting into smectite, intercrystalline micropores are rare, well H4, 2720.5 m, SEM; (**f**) Calcite cements completely occlude the intergranular pores, well H1, 2533.8 m, cross-polarized light; (**g**) Fibrous dawsonite, well H1, 2579.9 m, SEM; (**h**) framboidal aggregates of pyrite accompanied by illite and quartz overgrowth, well H2, 2582.5 m, SEM; (**i**) flaky chlorite growth on the edge of the quartz overgrowth, well H2, 2979 m, SEM; (**j**) dissolution of feldspar,co-precipitation of authigenic quartz, kaolinite and illite were observed within pore from feldspar dissolution. Note the illite cements were formed from illization of kaolinite, well H2, 2582.5 m, SEM. Qa = authigenic quartz, Qo = overgrowth, Cal = calcite, Daw = dawsonite, Mi = mica, F = feldspar, I = illite, K = kaolinite, Py = pyrite, Ch = chlorite, I/S = mixed layers of illite and smectite.

Sandstone of the K22 layer underwent mechanical compaction, as indicated by oriented grain arrangements (Fig. 8a) and deformed mica (Fig. 8b). Indications for chemical compaction, such as sutured contacts, are rare in this study interval.

#### Cementation

In the K22 sandstone, cementation minerals mainly include siliceous cements, clay minerals and carbonate cements. Siliceous cements are dominated by authigenic quartz, which is commonly found in intergranular pores adjacent to unstable grains, such as feldspar (Fig. 8c). In some cases, quartz overgrowth is observed.

The clay minerals (average 5.27%) are dominated by kaolinite, appearing as stacked booklets or vermiform aggregates (Fig. 8d). They are usually distributed in the residual primary intergranular pores or incompletely filled by other cements, such as calcite cements (Fig. 8d). Fibrous (Fig. 8e) or honeycomb-like illite with spiny terminations (Fig. 6d) is also abundant in the target sandstones. Stochastically oriented platelet-shaped chlorites mostly occur along the rims of grains (Fig. 8f). The relative contents of kaolinite, illite and chlorite range from 0 to 82%, 0–55%, and 0–68%, respectively.

Calcite (average 6.81%) is the most widely distributed carbonate cement in the Miocene Zhujiang Formation sandstones. In thin section, calcite completely plugging the intergranular pores is occasionally observed (Fig. 8g). However, in most cases, calcite cements partially fill the intergranular pores (Fig. 8d). Fibrous dawsonite aggregates are occasionally observed filling the intergranular pores (Fig. 8h).

Pyrite, as a minor cement, usually occurs in the form of framboidal aggregates (Fig. 8i). Point counting analysis shows that pyrite has a volume percentage from trace to 4.2%, averaging 1.2%.

#### Dissolution

Feldspar (Fig. 8j) and rock fragment (Fig. 6a,b) dissolution is the main secondary porosity factor. The photomicrographs of the thin sections show that feldspar is partially dissolved along the cleavage planes and fracture surfaces, leaving behind secondary intragranular pores (Fig. 6a). In some intensely dissolved intervals, moldic pores caused by the dissolution of rock fragments are observed (Fig. 6b).

## Discussion

### Diagenetic history

**Figure 9:**
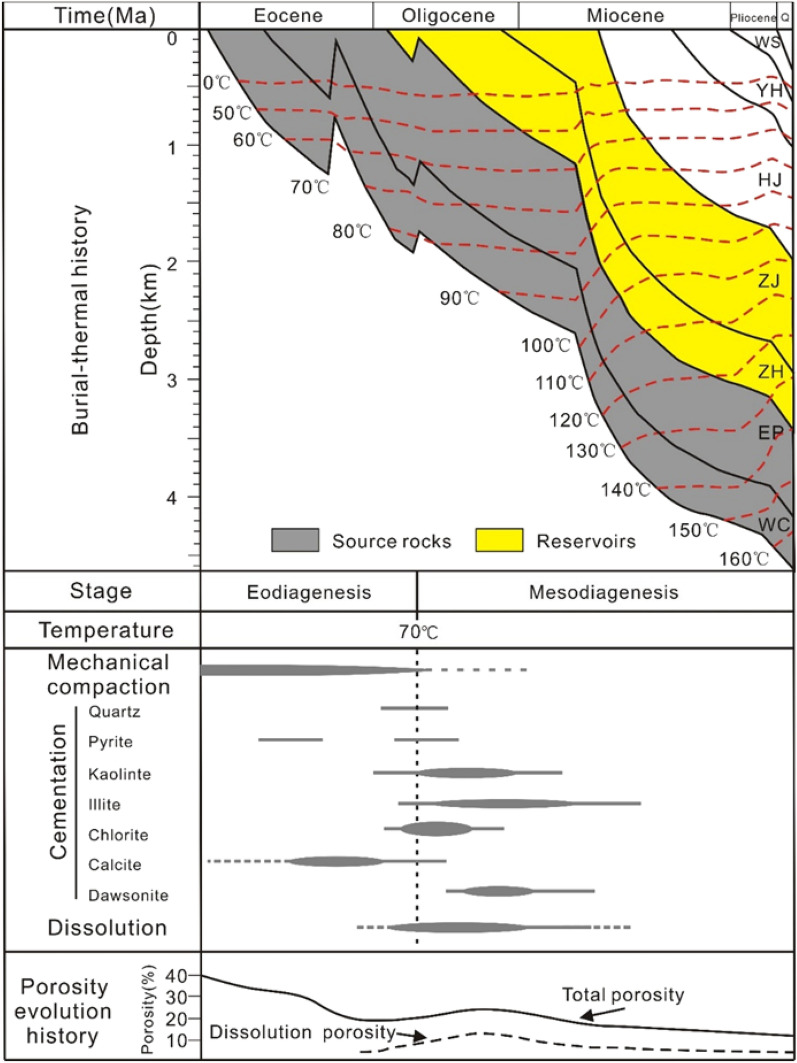
Burial-thermal history of Well H1 and the paragenetic sequence and types of diagenesis in the Miocene Zhujiang Formation sandstones^39,51^. Q = Quaternary.

The diagenetic history of the Miocene ZJ Formation sandstones is analysed according to the types of diagenetic processes, cements, pore types, physical properties and other aspects^37,38^. Based on thin section and SEM observations, the diagenetic processes of the studied interval consist of mechanical compaction; quartz; carbonate and clay mineral cementation; pyrite; and dissolution. According to Morad et al.^38^, the diagenetic processes can be divided into eodiagenesis and mesodiagenesis. Eodiagenesis in this area is characterized by sediments that underwent a paleogeotemperature of approximately 70 °C, which generally occurs at depths less than 2 km. As burial continues, the diagenetic process transitions to mesodiagenesis^38^. Based on the reconstruction of the burial-thermal history of well H1 in the Z21 oil–gas field by Wu^39^, it is found that the target ZJ Formation sandstones (mainly 2–3 km) have formation temperatures of 85–120 °C (Fig. 9), illustrating that mesodiagenetic processes occurred.

Mechanical compaction is the bulk volume reduction resulting from lithostatic stress, characterized by the reorientation of framework grains (Fig. 8a), deformation of ductile grains (Fig. 8b) or local fracturing of brittle grains (Fig. 6c). Mechanical compaction occurs simultaneously with sediment deposition and is considered to dominate under temperatures from 70 to 80 °C^40^, which mainly corresponds to the eodiagenetic stage. No chemical compaction is observed in thin sections.

Quartz cementation occurs in the eodiagenetic process, mainly in the form of authigenic quartz, which is mostly ascribed to abnormal formation pressure due to the injection of fluids such as CO_2_ from underlying strata. Mesodiagenetic quartz cementation in sandstones is often ascribed to intraformational dissolution of detrital silicate phases due to the low aqueous solubility of SiO_2_^41^. However, there is minimal pressure dissolution of detrital quartz in the ZJ Formation sandstones, suggested by no observed detrital quartz dissolution in thin sections and SEM. The typical temperatures of chlorite formation are approximately 60–70 °C^42^, which refers to the end of the eodiagenetic stage. The SEM photomicrographs show quartz overgrowths coated by authigenic chlorites, which indicates that authigenic chlorite occur after quartz overgrowths. The terminus of the quartz overgrowths are restricted by the occurrence of pyrite (Fig. 8i), indicating that pyrite occurs prior to quartz overgrowth. Feldspar dissolution is considered an important material resource for quartz, kaolinite and illite precipitation^43^; therefore, it is inferred that quartz, kaolinite and illite cementations occur penecontemporaneously or that kaolinite occurs slightly earlier than quartz cementations. Under acidic conditions, extensive illitization is associated with a temperature of 140 °C^44^ (but not limited to > 140 °C^45^), indicating that illite occurs in the eodiagenetic stage but is mainly formed in the mesodiagenetic stage.

The early calcite completely fills the intergranular pores, and the irregular shape of the calcite indicates that it is formed prior to or contemporaneous with severe mechanical compaction (Fig. 8g). In some cases, calcite, together with other clay minerals, such as kaolinite, usually partially fill the interparticle pores (Fig. 8d). Illite and kaolinite grow on the surfaces of calcite cementations, indicating that calcite precipitation occurs prior to both illite and kaolinite (Fig. 8d). Another kind of carbonate cementation, dawsonite, is considered an indicator of CO_2_^46,47^. The CO_2_ stored in the ZJ Formation sandstones in Z21oil-gas field was derived from organic matter evolution, together with the organic acids released from the lower EP and WC Formation^48^. The acid fluid interacts with detrital grains and diagenetic minerals, producing sufficient Na^+^ and Al^3+^ for dawsonite precipitation and resulting in secondary pores^49,50^. The dissolution of plagioclase (e.g., albite) after experiencing a mass influx of CO_2_ is generally accepted as a source mineral for dawsonite cements^46,47^. Therefore, dawsonite precipitation occurs after dissolution (Fig. 9).

### Controls on reservoir quality

#### Sedimentary controls on reservoir quality

**Figure 10:**
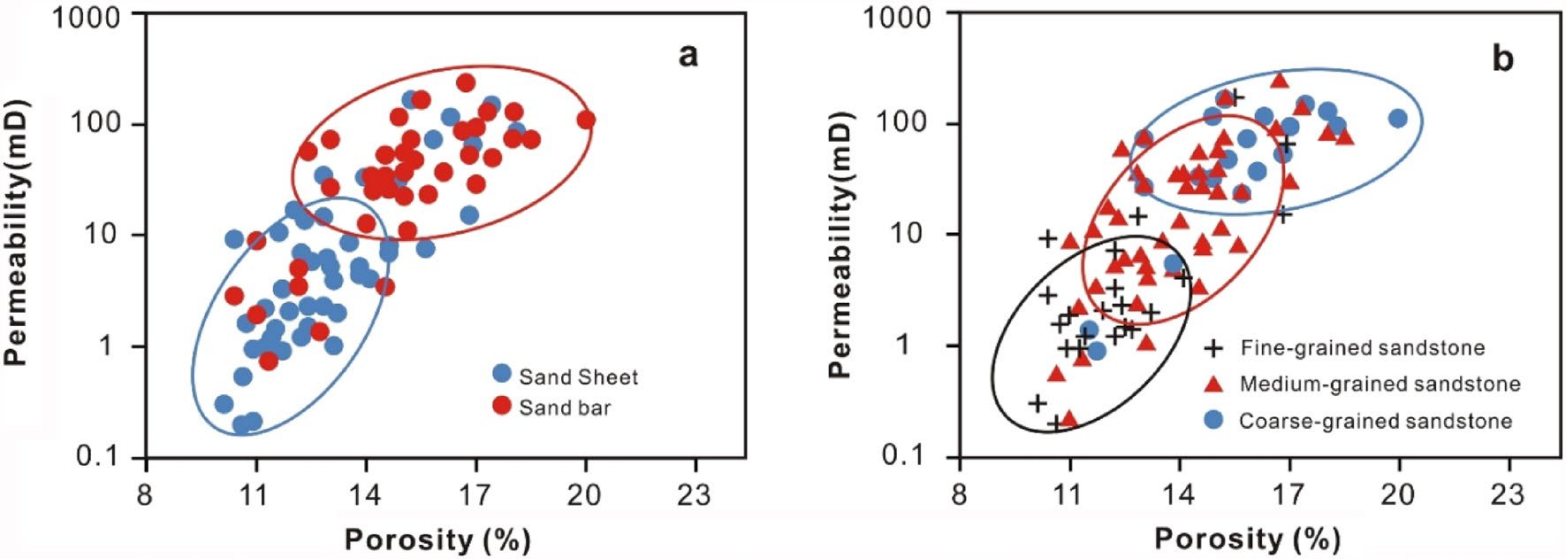
Statistics of porosity and permeability of different depositional elements (**a**) and different grain-sized sandstones (**b**).

1. *Depositional aspect* As mentioned above, deposits of the Z21 gas field were located far from the delta front, which is characterized by complex hydrodynamic conditions^10^ and variable sedimentary structures (Fig. 5). Linking the heterogeneous porosity and permeability (Fig. 7) to the varying lithofacies types, it is preliminarily inferred that the reservoir quality of the Z21 gas field was significantly affected by the depositional settings. As shown in Fig. 10a, statistics of different depositional systems show that both the porosity and permeability of SB are generally higher than those of SS. The SS depositional system is interpreted as sandstones deposited in a low-energy environment; they are poorly sorted and have a high matrix content^52^.
2. *Grain size controls on reservoir quality*. Another factor affecting the reservoir quality is grain size. The grain size, which reflects the primary texture of sandstones, may control the extent of the subsequent diagenetic events^53^. Statistics show that sandstones with different grain sizes, namely, fine-grained, medium-grained, and coarse-grained sandstones, have different porosity and permeability distribution centres (Fig. 10b). As the gain sizes increase, the porosity and permeability generally become larger (Fig. 10b). Compared to finer-grained sandstones, larger-grained sandstones are usually well sorted with fewer matrix grains; moreover, rigid framework grains such as quartz are less influenced by complex compaction processes if they are larger^54^.

#### Diagenetic controls on reservoir quality

**Figure 11:**
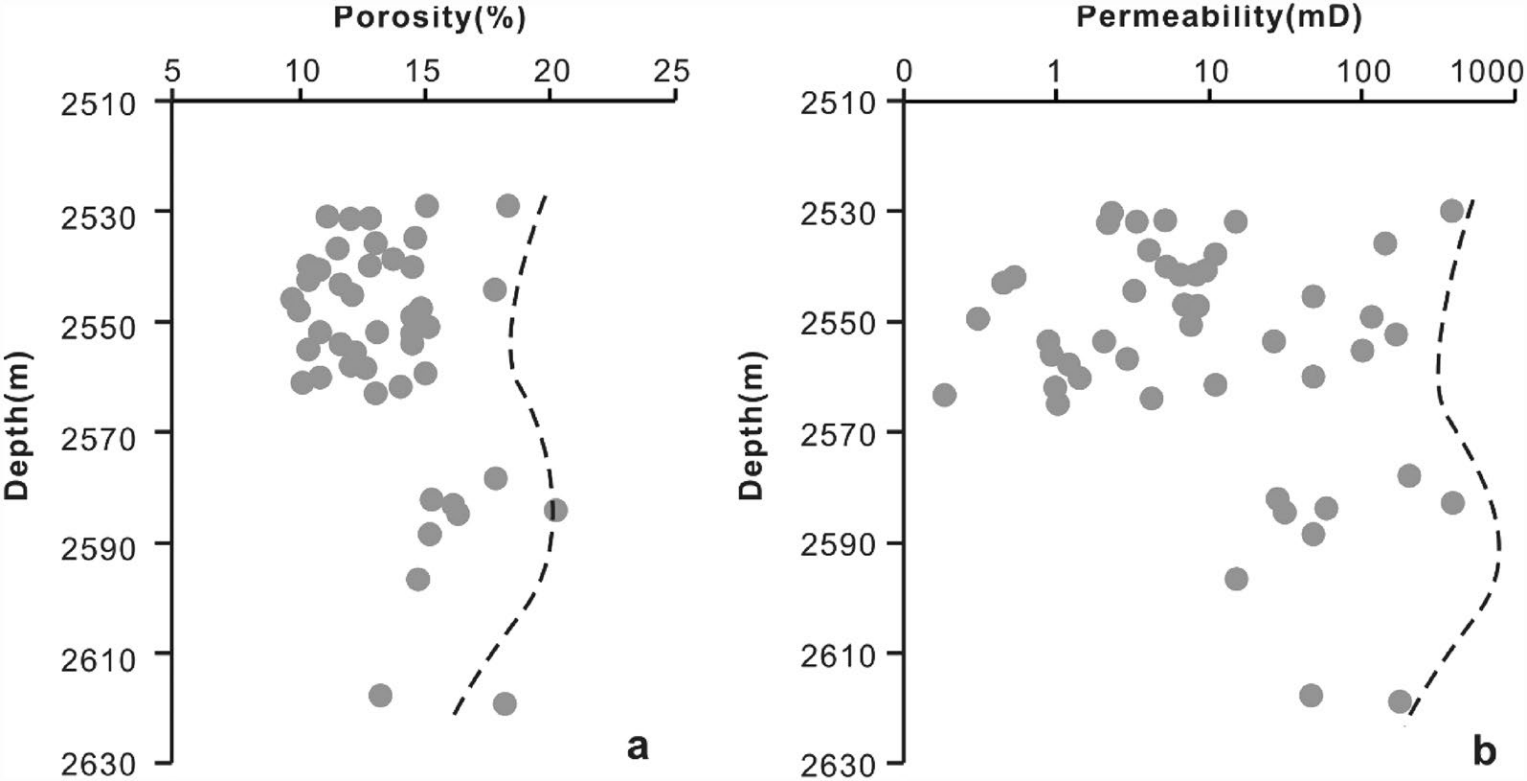
Plot of the depth versus porosity (**a**) and permeability (**b**) for the Miocene ZJ Formation sandstones of Z21 gas field in Huizhou Depression. The porosity and permeability are collected from Shenzhen Branch of CNOOC (China National Offshore Oil Corporation).

**Figure 12:**
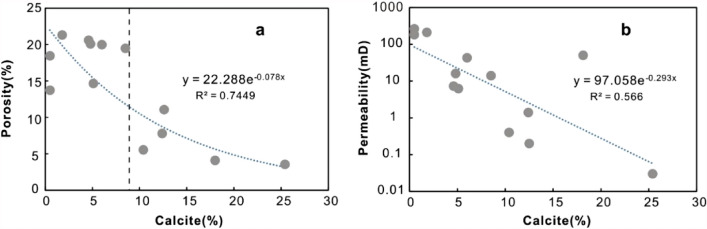
Plot of the calcite versus porosity (**a**) and permeability (**b**) for the Miocene ZJ Formation sandstones of Z21 oil–gas field field in Huizhou Depression. The calcite content was obtained from point-counting on thin sections.

**Figure 13:**
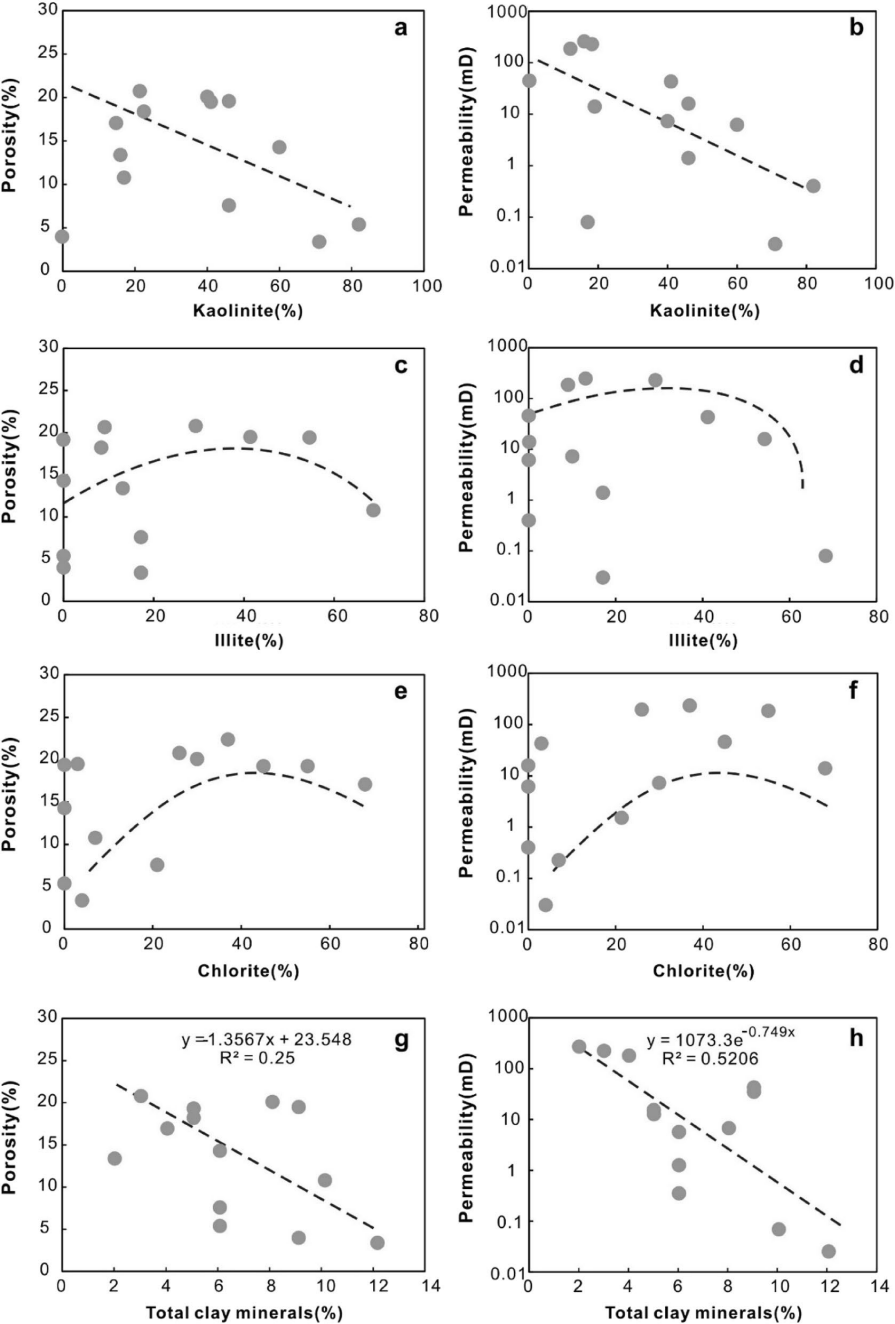
Variations in the relative content of kaolinite (**a,b**), illite (**c,d**), chlorite (**e,f**) and total content of clay minerals (**g,h**) with the porosity and permeability. The relative mineralogical composition of each clay minerals was quantitatively analyzed via X-ray diffraction (XRD).

**Figure 14:**
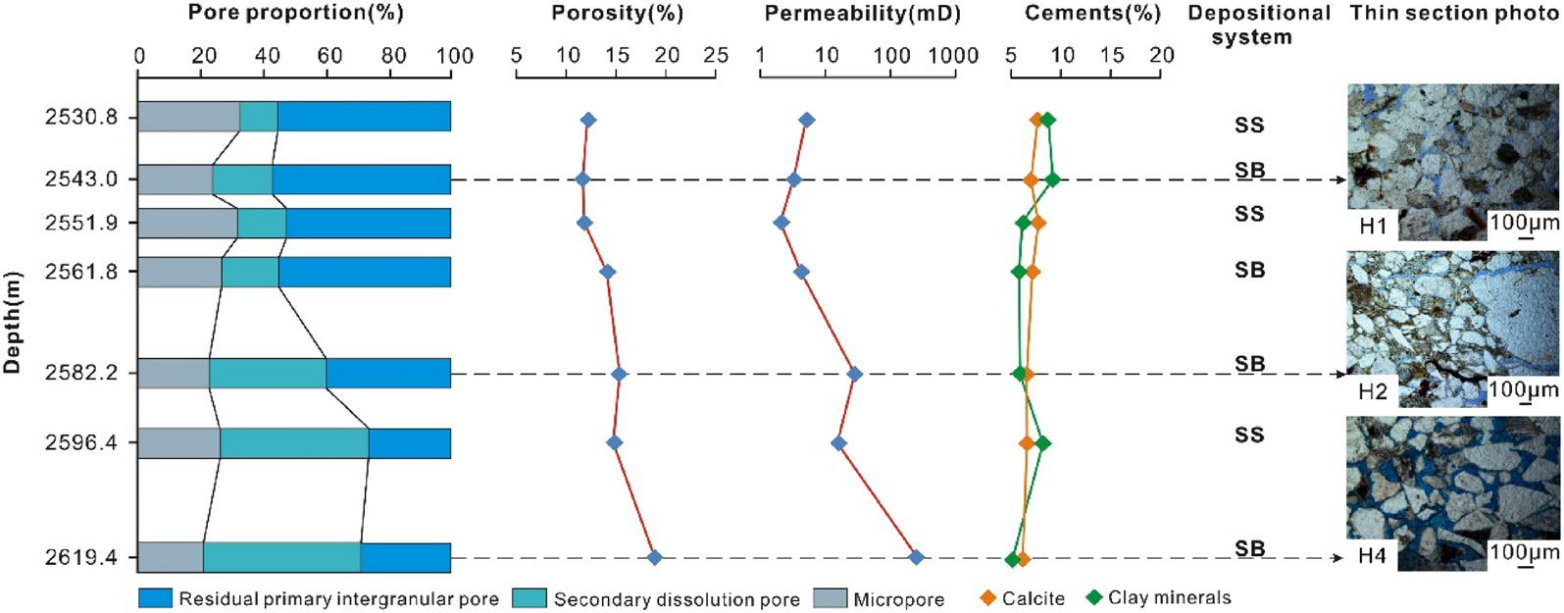
Tendency chart between pore proportion and porosity, permeability, cements and depositional systems of certain samples.

1. *Mechanical compaction* Mechanical compaction, intergranular pressure solution, cementation, framework grain dissolution, and cement dissolution have all been documented as playing significant roles in modifying the porosity of various sandstones^55^. In the Miocene ZJ Formation sandstones, the mechanical compaction is characterized by the directional arrangement of grains, concave-convex contacts between the grains and plastic deformation of ductile grains. Upon burial, sediments compact mechanically when the effective stress due to overburden is increased, so that the porosity and the total rock volume are reduced^56^. As a result of increasing effective stress from the overlying strata during burial, the effect of mechanical compaction increases with increasing burial depth in eodiagenesis^57^. As shown in Fig. 11, as the burial depth increases (2530–2570 m and 2585–2620 m), both porosity and permeability decrease with depth. Why do the porosity and permeability increase as the reservoir deepens in the 2570–2585 m interval? This result can likely be ascribed to two main aspects. First, there is not sufficient sample data related to this depth range. Second, at burial depths greater than approximately 2 km (> 70–80 °C), quartz precipitation on clastic grains (Fig. 8f) gradually produces a framework of quartz overgrowths that are strong enough to prevent further mechanical compaction^56^, and dissolution of feldspar enhances the porosity volume, as indicated by Fig. 8j.
2. *Cementation* Carbonate cements in this area are dominated by calcite. Dawsonite is ignored for quantitative statistical analysis due to limited samples encountering dawsonite cements. Calcite partially (Fig. 8d) or completely (Fig. 8g) fills intergranular pores, and both reduce the pore spaces. As shown in Fig. 11, in general, both porosity and permeability decrease with increasing calcite content, showing a remarkable negative relationship with R^2^ = 0.7449. However, when the content of calcite is less than 9%, there is no remarkable negative correlation between porosity and calcite (Fig. 12a). The sample has a relatively higher calcite content of 18% with a permeability value of 48 mD (Fig. 12b) and a porosity value of 4% (Fig. 12a), and the sample is interpreted by considering the development of microfractures (Fig. 6c).
  Typically, authigenic kaolinite, illite or other clay minerals may be found in nearby primary or secondary pores^58^. Precipitation of kaolinite can occur only when the K^+^/H^+^ ratio and silica concentration in the pore water are below certain values, and such low K^+^/H^+^ ratios are normally found only in fresh or brackish water^59^. This means that in intervals where the CO_2_ concentration is high and the dissolution of feldspar and debris is severe, the relative content of kaolinite precipitation could reach 82% (Table 2). However, the intercrystalline micropores within kaolinite aggregates are poorly developed. Therefore, the more kaolinites there are, the more pore spaces that are filled, showing a negative correlation between kaolinite and reservoir physical properties (Fig. 13a,b). For illite, intercrystalline micropores within illite aggregates are generally well developed (Fig. 5d), the porosity and permeability increase as the illite content increases (Fig. 13c,d). However, the conversion from illite to mixed layers of illite and smectite reduces some of the pore spaces (Fig. 8e). On the other hand, the pore-filling illite aggregates may occupy some pore spaces and result in a decrease in porosity and permeability (Fig. 13c,d).
  Grain-coating chlorites are generally considered porosity-preserving components in sandstones^42,52,59^. Within the ZJ Formation, as shown in Fig. 13e, the porosity increases and then decreases slightly, implying that the chlorite coatings may retard quartz overgrowths within a limited content range (Fig. 8f). The relationship between chlorite and permeability is similar to that between chlorite and porosity (Fig. 13f). As the volume of chlorite accumulates, the porosity and permeability decrease slightly due to the plugging of pore-filling chlorite aggregates.
  In the study area, although a single type of clay mineral may enhance the reservoir physical properties, another may worsen the congeneric properties. The porosity and permeability plots (Fig. 13g,h) still display a decreasing trend with the total clay minerals (low R^2^ values: 0.25 and 0.5206, respectively), indicating that the clay mineral matrix is an important factor controlling reservoir quality in the area.
3. *Dissolution* Dissolution is generally considered a constructive factor that enhances reservoir quality^52,58,59^. The secondary dissolution pores are dominated by the dissolution of feldspar and debris, and therefore, it is meaningful to ascertain whether dissolution is responsible for the deeper sandstones but with higher porosity and permeability. The types of pore reflected by thin sections were analysed by point counting. The results show that the deeper sandstones with higher porosities and permeabilities usually have a greater proportion in secondary dissolution pores, whereas the shallower sandstones are dominated by residual primary intergranular pores, which are limited in bulk volume (Fig. 14). Although the influence of dissolution on reservoir quality studied in this way is not very rigorous, considering the limited impact from cements and depositional systems (Fig. 14), to some extent, dissolution enhancing the porosity and permeability is still proven qualitatively.

**Table 2:** Relative content of the clay minerals via XRD and the porosity and permeability of the selected samples.

Well	Depth(m)	Total clay (%)	Relative content of clay minerals (%)				Ф (%)	K (mD)
			K	I	I/S	C		
H3	2581	6	60	0	40	0	14.30	6.20
H3	2581.5	8	40	10	20	30	20.10	7.30
H3	2582	6	82	0	18	0	5.40	0.40
H3	2582.3	5	46	54	0	0	19.60	16.00
H3	2582.5	9	0	0	55	45	4.00	46.00
H3	2579.9	5	19	0	13	68	19.00	14.00
H3	2702.8	9	41	41	15	3	19.50	43.00
H2	2429.7	6	46	17	16	21	7.60	1.40
H2	2431.7	10	17	68	8	7	10.80	0.20
H2	2436.6	3	18	29	27	26	20.80	210.20
H2	2433.8	12	71	17	8	4	3.40	0.03
H6-1	2036.54	2	16	13	34	37	13.40	230.00
H6-1	2039.9	4	12	9	24	55	18.00	186.00

*K* kaolinite, *I* illite, *I/S* mixed layers of illite and smectite, *C* chlorite, *Ф* measured porosity, *K* measured permeability.

## Conclusions

The Miocene sandstones are composed of medium-grained, moderately sorted subarkose and lithic arkose with an average framework composition of Q_76_F_14_R_10_. The sandstones are characterized by low porosity (average 13.9%) and low permeability (average35.8 mD), forming poor-quality reservoir.

A total of 6 depositional lithofacies, namely, Sp, Sm, Sr, Sw, Sl, and Fl, and two depositional systems, namely, SB and SS, were identified. SB was deposited in a relatively high-energy hydrodynamic environment. Generally formed coarser-grained sandstone and forming better-quality reservoirs than in SS.

Mechanical compaction and cementation of calcite are responsible for the porosity and permeability reduction. The clay minerals such as grain-coating chlorites may enhance the preservation of porosity within a certain content range; however, the total clay mineral cementation is not considered as an important factor of reservoir quality. Dissolution of detrital grains has enhancing effect on secondary porosity.
